# Validation and extension of the reward-mountain model

**DOI:** 10.3389/fnbeh.2013.00125

**Published:** 2013-10-01

**Authors:** Yannick-André Breton, Ada Mullett, Kent Conover, Peter Shizgal

**Affiliations:** Department of Psychology, Groupe de Recherche en Neurobiologie Comportementale, Center for Studies in Behavioural Neurobiology, Concordia University Montréal, QC, Canada

**Keywords:** intracranial self-stimulation, brain stimulation reward, temporal integration, medial forebrain bundle

## Abstract

The reward-mountain model relates the vigor of reward seeking to the strength and cost of reward. Application of this model provides information about the stage of processing at which manipulations such as drug administration, lesions, deprivation states, and optogenetic interventions act to alter reward seeking. The model has been updated by incorporation of new information about frequency following in the directly stimulated neurons responsible for brain stimulation reward and about the function that maps objective opportunity costs into subjective ones. The behavioral methods for applying the model have been updated and improved as well. To assess the impact of these changes, two related predictions of the model that were supported by earlier work have been retested: (1) altering the duration of rewarding brain stimulation should change the pulse frequency required to produce a reward of half-maximal intensity, and (2) this manipulation should not change the opportunity cost at which half-maximal performance is directed at earning a maximally intense reward. Prediction 1 was supported in all six subjects, but prediction 2 was supported in only three. The latter finding is interpreted to reflect recruitment, at some stimulation sites, of a heterogeneous reward substrate comprising dual, parallel circuits that integrate the stimulation-induced neural signals.

## Introduction

Intracranial self-stimulation (Olds and Milner, 1954) has played an indispensable role in the study of the neural circuitry underlying valuation and goal selection. This phenomenon captures many of the features of performance for natural rewards (Green and Rachlin, 1991; Conover and Shizgal, 1994) while offering superior experimental control. The subjects do not become satiated from consuming the electrical reward, and they will work tirelessly when the reward is strong; both the timing of reward delivery and the strength of the rewarding effect can be adjusted with precision. The rewarding effect arises from observable neural activity induced by the stimulation in the vicinity of the electrode tip, a valuable feature for tracing brain reward circuitry and identifying its components.

The selection and pursuit of rewards arise from the interaction of multiple psychological processes and neural systems (Gallistel, 1983; Robbins and Everitt, 1996; Balleine and Dickinson, 1998; White and McDonald, 2002; Berridge and Robinson, 2003). Among these are the processes and systems responsible for extraction and storage of information about the strength and cost of rewards. The intracranial self-stimulation paradigm lends itself particularly well to the isolation and study of these attributes of rewards, as well as others, such as delay and probability. However, there has been considerable controversy about how best to measure the effects of manipulating these attributes and about the inferences that can be drawn from such behavioral measurements.

In the initial studies of intracranial self-stimulation, response-rates were used to measure changes in the effectiveness of the stimulation in producing reward-seeking behavior (Olds, 1958). However, serious reservations were soon advanced concerning the use of response rate as a measure of reward efficacy (Hodos and Valenstein, 1962). Curve-shift (Edmonds and Gallistel, 1974, 1977; Miliaressis et al., 1986) and progressive-ratio (Hodos, 1961) measures were introduced to address these reservations and have been used extensively to quantify the effects of lesions and pharmacological agents (Keesey and Goldstein, 1968; Edmonds and Gallistel, 1977). The curve-shift method entails measurement of operant performance as a function of the strength of the rewarding stimulation, whereas the progressive-ratio method entails measurement of operant performance as a function of reward cost. Although much progress has been achieved by means of these methods, recent work reveals a fundamental ambiguity in curve-shift and progressive-ratio measurements (Arvanitogiannis and Shizgal, 2008; Hernandez et al., 2010). One source of this ambiguity can be removed by application of a new method that entails measurement of performance as a function of both the strength and cost of reward. The resulting three-dimensional (3D) structure has been dubbed the “reward mountain” (Arvanitogiannis and Shizgal, 2008; Hernandez et al., 2010).

A key advantage of the reward-mountain method is that it isolates changes in the sensitivity of brain reward circuitry from a diverse collection of other variables that also contribute to reward pursuit. These variables include reward-circuit gain, subjective effort cost, and the value of activities, such as resting, grooming, and exploring, that compete with pursuit of the reward offered by the experimenters. The sensitivity of the neural circuitry underlying brain stimulation reward (BSR) is estimated from the pulse frequency required to produce a rewarding effect of given magnitude, whereas the gain of this circuitry determines the maximum rewarding effect that can be achieved. The ability of the reward-mountain method to distinguish sensitivity changes from changes in gain, subjective effort cost, and the value of alternate activities has been exploited to shed new light on the stage(s) of processing at which psychomotor stimulants (Hernandez et al., 2010, 2012), neuroleptics (Trujillo-Pisanty et al., 2012), and cannabinoids (Trujillo-Pisanty et al., 2011) influence pursuit of BSR. However, the initial study validating the reward-mountain method (Arvanitogiannis and Shizgal, 2008) employed somewhat different procedures than those in force in the subsequent work. The current study was undertaken to assess the newer variant of the method and an extension of the most recent version of the model (Hernandez et al., 2010).

In the initial reward-mountain study (Arvanitogiannis and Shizgal, 2008), a variable-interval (VI) schedule of reinforcement was in effect. The 3D structure was built from four two-dimensional (2D) components, dubbed “sweeps.” Two of these consisted of response-rate measurements carried out sequentially at each element of a descending series of pulse frequencies, with either a short or long VI value. The remaining two sweeps consisted of response-rate measurements carried out at each element of an ascending series of VI values, with either a low or a high pulse frequency. Multiple determinations of a given sweep type were performed before moving on to the next. In contrast, in the pharmacological studies carried out using the 3D method (Hernandez et al., 2010, 2012; Trujillo-Pisanty et al., 2011, 2012), a “cumulative handling-time” schedule of reinforcement (Breton et al., 2009b) was in effect. This schedule replaces the VI with a fixed opportunity cost (time required to harvest a reward, dubbed the “price” of the reward). This change was introduced to reduce the subject's uncertainty about the reinforcement contingencies and to provide tighter experimental control over reward cost. Subsequent to the initial study, we found that repeated testing at a constant price reduced the evaluability of this reward-cost variable (Breton et al., 2009b). Thus, whenever feasible in subsequent studies, multiple sweep types have been intermixed in each test session, or predefined vectors of pulse frequencies and prices have been sampled randomly. To reduce the time required to survey the mountain, the number of sweep types has been reduced from four to three.

An additional procedural modification is the use of resampling-based methods (Efron and Tibshirani, 1994) to fit the mountain model to the data. The power and robustness of these methods increase the sensitivity with which shifts in the position of the mountain can be detected.

We now report an extension of the reward-mountain model (derived in the Appendix) that incorporates more realistic assumptions and recent data concerning: (1) the translation of objective opportunity costs into their subjective equivalents (Solomon et al., 2007), and (2) the frequency-following capabilities of the directly stimulated neurons responsible for the rewarding effect (Simmons and Gallistel, 1994; Solomon et al., 2010). These extensions helped us retest a key prediction of all versions of the mountain model: that changing the duration of the stimulation train should shift the mountain along the pulse-frequency axis. This prediction arises from the fact that the rewarding effect grows over the course of a stimulation train (Gallistel, 1978; Sonnenschein et al., 2003). If the train is of brief duration, the rewarding effect must grow very quickly in order to reach a substantial level by the end of the train. A high stimulation strength is required to achieve this. However, if the train is of longer duration, the rewarding effect can grow more gradually and still reach the same final level. Thus, lower stimulation strength can suffice to produce the same final level of reward intensity at the end of a long-duration train as achieved by delivering higher-strength stimulation during a shorter-duration train. This prediction was borne out using the earlier procedures (Arvanitogiannis and Shizgal, 2008) and was confirmed in the present study. We also retested a second prediction, one that depends on the assumption that a unitary circuit is responsible for the temporal integration of the rewarding effect. Given that assumption, the reward-mountain should not shift systematically along the price axis in response to a change in train duration. This prediction was also borne out in the initial study (Arvanitogiannis and Shizgal, 2008), but the more sensitive methods employed here reveal that this prediction holds only at some stimulation sites. The violation of this prediction may reflect the recruitment, at other stimulation sites, of heterogeneous reward circuitry in which parallel components perform temporal integration of the stimulation-induced reward signal.

## Methods

### Surgery and training

Five Long-Evans rats (Charles River, St-Constant, QC) weighing a minimum of 350 g before surgery, were implanted unilaterally with an electrode made from a 00 insect pin coated with Formvar enamel to within 0.5 mm from the tip. Surgery was performed under isofluorane anesthesia at a 3% concentration. Atropine sulfate (0.05 mg/kg) was administered prior to surgery, and buprenorphine (0.05 mg/kg) was administered immediately following surgery as well as 24 and 48 h post-operatively. Electrodes were aimed at the left, lateral hypothalamic level of the medial forebrain bundle, at the following level-skull stereotaxic coordinates: 2.8 mm posterior to bregma, 1.7 mm lateral to the midline, 9 mm ventral to the skull surface.

A fixed, cumulative handling-time schedule of reinforcement was in effect throughout the experiment. As described in Breton et al. (2009b), this schedule delivers a reward once the lever has been held for a cumulative amount of time (the “price” of the reward). Each downward and upward transition of the lever during the trial was time-stamped and recorded. The lever was retracted upon the triggering of the rewarding stimulation and then re-extended into the test cage following a 2 s “blackout” delay.

In the current study the price was fixed throughout a trial but could vary from one trial to the next so as to alter the opportunity cost of the electrical reward. (An opportunity cost is the benefit that was forgone by devoting time to pursuit of the electrical reward rather than to alternate activities such as grooming, resting, or exploring). The strength (pulse frequency) of the stimulation was also held constant during each trial but could vary from one trial to the next so as to alter the intensity of the rewarding effect. The duration of each trial was set so as to allow the rat to obtain 25 rewards if it held the lever down during the entire time it was extended into the test cage. Thus, trial duration covaried with the price of the stimulation.

When the trial duration timed out, the lever was retracted, the cue light was extinguished, and a 10 s inter-trial interval began, during which an orange house light flashed. Two seconds before the end of the inter-trial interval, a single 0.5 s stimulation train was delivered non-contingently. The pulse frequency of this “priming” train was set to the maximum value to be used in the experiment and was held constant throughout the experiment. The priming stimulation provided no information about the pulse frequency that would be in force during the subsequent trial, but it helped motivate the rat to resume working.

Following screening for electrode efficacy, animals were trained on descending pulse-frequency sweeps at a 1 s price, and on ascending price sweeps at the highest frequency that did not produce disruptive side-effects, such as forced movements. Rats C17, C26, and Y12 were then presented with a descending pulse-frequency sweep composed of trial triads. Within a triad, each test trial was preceded by a leading “bracket” trial on which the pulse frequency was set to the maximum tolerable value, the train duration was 0.5 s, and the price was 1 s. The test trial was followed by a trailing bracket trial on which the pulse frequency was set to a value too low to support sustained responding, the train duration was 0.5 s, and the price was 1 s. This bracketed trial structure was also used in ascending price sweeps obtained at the highest tolerable pulse frequency. On test trials in all types of sweeps, the train duration was 0.5 s. Rats Y13, Y14, and Y15 were not trained on these “bracketed” sweeps. Instead, they entered the stabilization phase described below immediately following training on “unbracketed” sweeps. In the cases of these three rats, the bracketing procedure was introduced at the onset of the stabilization phase. From that point onward, the same procedures were used with all subjects.

The bracket trials served two purposes during the experiment. First, they provided stable reference points for the payoffs on offer during the test trials. Second, they provided objective evidence of whether performance remained stable over the duration of the test sessions.

### Behavioral testing

On the basis of preliminary fits of the mountain model to the data from the initial bracketed sweeps (in the case of rats C17, C26, and Y12) or training frequency and price sweeps (in the case of rats Y13, Y14, and Y15), we compiled two sets composed of three lists of pulse frequencies and prices, one set for each of the train durations (0.25 s and 1 s). These lists specify the pulse-frequencies and prices for the test trials (the middle trial of each triad). The values in the lists were chosen so as to survey the mountain structure efficiently. When viewed in a 2D space with one logarithmic axis representing the pulse frequency and the second logarithmic axis representing the price, the values in each of the lists correspond to nine equally spaced points along a straight line, dubbed a “pseudo-sweep.” One line (the pulse-frequency pseudo-sweep) runs parallel to the pulse-frequency axis, at a low price. A second line (the price pseudo-sweep) runs parallel to the price axis, at a high frequency. The third line (the radial pseudo-sweep) runs diagonally downwards from a high pulse-frequency, low-price value to a low pulse-frequency, high-price value. The radial pseudo-sweep was positioned so as to run through the point defined by the two location parameters of the mountain model: *F*_*hm*_, the pulse frequency at which the intensity of the reward is half-maximal, and *P*_*e*_, the price at which allocation of time to pursuit of a maximal reward is half-way between its minimal and maximal values. The values composing the pseudo-sweeps were chosen to maximize the likelihood of obtaining 3 points for which performance would be asymptotically high, 3 points for which it would be asymptotically low, and 3 points along the rising portion of each set. Due to the expectation that non-asymptotic performance would be highly variable, the 5 central price-frequency pairs of each set were sampled twice as often as those in the upper and lower extremes.

Following completion of training, the stabilization phase began. Bracketing was introduced for rats Y13, Y14, and Y15 at this stage. Otherwise, conditions were identical to those in force during the preliminary testing, except that the element of the six pseudo-sweeps in force on a given test trial was chosen randomly, without replacement, until all values had been sampled. A single pass through all six pseudo-sweeps is called a “survey” of the reward mountains for the short and long train durations. Each element of a pseudo-sweep specifies a pulse frequency, price, and train duration. In effect, the elements of all six pseudo-sweeps were combined in a virtual urn, and a sample was drawn at random on each test trial until the urn was empty, thus completing a survey.

Performance was deemed stable when time allocation was consistently high on leading bracket trials and consistently low on trailing bracket trials. When necessary, the pulse-frequency and price components of the pseudo-sweeps were adjusted so that time allocation varied as described above: 3 points with asymptotically high performance, 3 points with asymptotically low performance, and 5 points (sampled twice as often) within the dynamic range, for each pseudo-sweep. Typically, five surveys were required to achieve stable performance (3 for Y13; 4 for C17; 5 for C26, Y12, and Y14; and 6 for Y15). The main and final phase of the experiment then commenced.

Daily sessions were restricted to a duration of 4 h, during the dark phase of the 12/12 h light-dark cycle. Between 3 and 4 test sessions were required to complete a survey of the reward mountains. During the final phase of the experiment, each rat ran through 8 complete surveys, during which no further changes were made to the list of prices and frequencies to be presented at each train duration.

## Results

The dependent measure was time allocation (TA), the proportion of trial time, excluding the 2 s blackout delay, that the rat spent working for brain stimulation rewards. The time before the first reward was delivered was excluded from TA because during that time, the rat does not yet have information about the strength and cost of the reward on offer. Work time included both the total time the lever had been depressed and the sum of the times the lever was released for less than 1 s. These short interruptions of lever depression (“taps”) were included because when they occur, the rat is very near the lever, actively pursuing rewards (Breton et al., 2009b). Independent variables used in the analyses were the train duration [long (1 s) or short (0.25 s)], the pulse frequency (the number of current pulses delivered per second in each stimulation train), and the price (the cumulative time the lever had to be depressed in order to trigger delivery of a reward).

### The extended reward-mountain model

The reward-mountain model was fit to the data obtained during the final phase of the experiment. The surfaces fit to the results obtained during these 8 surveys are defined by an extension of an earlier version of the reward-mountain model (Hernandez et al., 2010). The expression used in that paper is:

(1) $$TA=\left[\left(TA_{\max}-TA_{\min}\right)\times \frac{{\left(\frac{F^{g}}{F^{g}+F_{hm}^{\;g}}\right)}^{a}}{{\left(\frac{F^{g}}{F^{g}+F_{hm}^{\;g}}\right)}^{a}+{\left(\frac{P}{P_{e}}\right)}^{\text{​}a}}\right]+TA_{\min}$$

where

$$\begin{array}{l} \;a=\text{the payoff-sensitivity exponent}\\ \;F=\text{the pulse frequency}\\ \;F_{hm}=\text{the pulse frequency at which reward intensity is half maximal}\\ \;g=\text{the reward-growth exponent}\\ \;P=\text{the price of the stimulation train}\\ \;P_{e}=\text{the price at which time allocation for a maximally intense reward falls halfway between }TA_{\max}\text{ and }TA_{\min}\\ TA_{\max}=\text{the maximal time allocation}\\ TA_{\min}=\text{the minimal time allocation} \end{array}$$

According to Equation 1, the subjective price is the same as the objective value, and the stimulated neurons fire once per pulse regardless of the pulse frequency. Both of these assumptions break down at extremes (Solomon et al., 2007, 2010). Given the wide range of prices and pulse frequencies employed in this study, Equation 1 was extended to accommodate measurements of the form and parameters of the functions relating subjective to objective prices (Solomon et al., 2007) and firing frequencies to pulse frequencies (Solomon et al., 2010). The resulting equation is as follows:

(2) $$TA=\left[\left(TA_{\max}-TA_{\min}\right)\times \frac{{\left(\frac{FF^{g}}{FF^{g}+FF_{hm}^{\;g}}\right)}^{\text{​}a}}{{\left(\frac{FF^{g}}{FF^{g}+FF_{hm}^{\;g}}\right)}^{a}+{\left(\frac{SP}{SP_{e}}\right)}^{a}}\right]+TA_{\min}$$

where

$$\begin{array}{l} \;FF=\text{the induced frequency of firing in the directly-stimulated neurons}\\ FF_{hm}=\text{the induced frequency of firing that produces a rewarding effect of half-maximal intensity}\\ \;SP=\text{the subjective price of the stimulation train}\\ \;SP_{e}={\text{the subjective price at which time allocation for a maximally intense reward falls halway between TA}}_{\min}\text{ and }TA_{\max} \end{array}$$

The expression that translates objective into subjective prices is

(3) $$SP=SP_{\min}+SP_{\text{bend}}\times \text{Ln}\left(1+e^{\frac{P-SP_{\min}}{SP_{\text{bend}}}}\right)$$

where

$$\begin{array}{l} \;SP_{\min}=\text{minimum subjective price}\\ SP_{\text{bend}}=\text{parameter determining the abruptness of the transition between }SP_{\min}\text{ and the rising portion of the subjective price function} \end{array}$$

**Figure 1:**
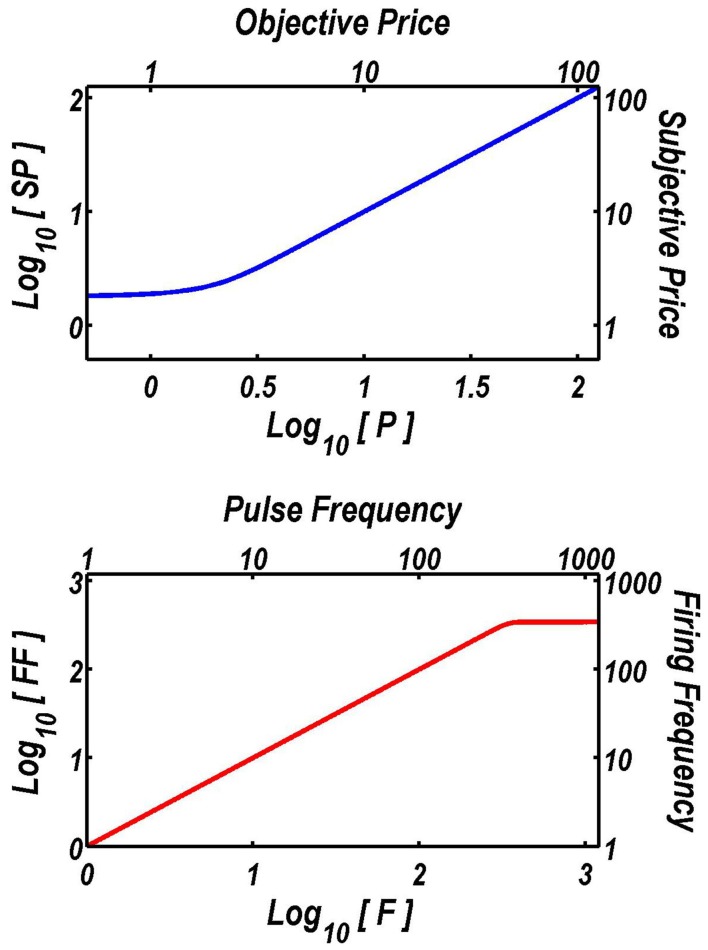
**Extensions of the reward-mountain model**. The upper graph shows the function that translates objective prices into subjective ones. The form and parameters of this “subjective-price” function are based on an experiment by Solomon et al. (2007). When prices are very low, changes in their objective value have little subjective impact. In contrast, once the price exceeds about 4 s, the subjective price converges on the objective price. The lower graph shows the function that translates the pulse frequency into the induced frequency of firing in the directly stimulated neurons subserving the rewarding effect of the electrical stimulation. The form and parameters of this “frequency-following” function are based on another experiment by Solomon et al. (2010). As long as the pulse frequency is lower than about 340 pulses per second, each stimulation pulse triggers an action potential in the directly stimulated neurons. The frequency-response function then levels off quite abruptly, and increasing the pulse frequency further has little effect.

On the basis of prior experiments (Solomon, in preparation; Solomon et al., 2007), *SP*_min_ was set to 1.75 s, and *SP*_bend_ to 0.57 s. The subjective price (SP) approaches *SP*_min_ when the objective price (*P*) is very low, and it converges on *P* as *P* grows (upper panel of Figure 1). Given the parameter values employed in the fits, the subjective price is within 0.2% of the objective price once *P* equals 4 s.

The expression that translates pulse frequencies into firing frequencies is

(4) $$FF=F_{\text{bend}}\times \left[\text{Ln}\left(1+e^{\frac{F_{\text{NearMax}}}{F_{\text{bend}}}}\right)-\text{Ln}\left(1+e^{\frac{F_{\text{NearMax}}-F}{F_{\text{bend}}}}\right)\right]$$

where

$$\begin{array}{l} \;F_{\text{bend}}=\text{parameter governing the abruptness of the transition between the rising and flat segments of the function}\\ F_{\text{NearMax}}=\text{the midpoint of the transitional region} \end{array}$$

On the basis of prior experiments (Solomon, in preparation; Solomon et al., 2010), *F*_bend_ was set to 20.63 pulses per s (pps), and *F*_NearMax_ was set to 342.9 pps. Given these values, the induced frequency of firing in the directly stimulated neurons asymptotes near 343 pps (lower panel of Figure 1). The values of *F*_bend_ and *F*_NearMax_ are broadly compatible with earlier results (Simmons and Gallistel, 1994).

### Resampling and fitting procedure

Equation 2 was fitted separately to the time-allocation data from each rat so as to generate one reward mountain for the short train duration and another for the long. In order to limit the number of free parameters and to focus the analysis on the predictions we had set out to test, the only parameters free to vary across train duration conditions were *F*_*hm*_, *P*_*e*_, and *TA*_max_. Thus, the two mountains for each rat share a common floor as well as common slopes but mountains can differ in altitude and in their locations within the space defined by the strength and price of the stimulation. Allowing *TA*_max_ to vary across train duration conditions was deemed appropriate because the stimulation tended to produce more severe disruptive motoric side-effects at the longer train duration than at the shorter one.

A bootstrapping approach (Efron and Tibshirani, 1994; Hernandez et al., 2010) implemented in MATLAB (The Mathworks, R2012b) was used to estimate *P*_*e*_, *F*_*hm*_, and *TA*_max_ as well as to compute the associated confidence intervals. This procedure entails drawing multiple samples from the original data, with replacement.

Each point along a pseudo-sweep is defined by the combination of a train duration, pulse frequency and price. Either 8 or 16 measurements of time allocation were obtained at each of these points (16 for the central 5 points and 8 for the 2 points at either end). One thousand samples, each consisting of the same number of values as the original sample (8 or 16), were drawn at random and with replacement from the original data at each point along each of the 6 pseudo-sweeps. The surface described by Equation 2 was then fitted to each of the resulting 1000 datasets. This generated 1000 estimates of *P*_*e*_, *F*_*hm*_, and *TA*_max_ for each train duration and 1000 estimates of *a*, *g*, and *TA*_min_ for both train durations together. The means of each set of 1000 parameter estimates were computed along with corresponding 95% confidence regions defined as the range excluding the lowest and highest 25 estimates of the 1000 generated. The criterion for a statistically reliable shift in a location parameter was an absence of overlap in the 95% confidence regions about the estimates of *F*_*hm*_ or *P*_*e*_ for the two train durations.

### Fitted surfaces

**Figure 2:**
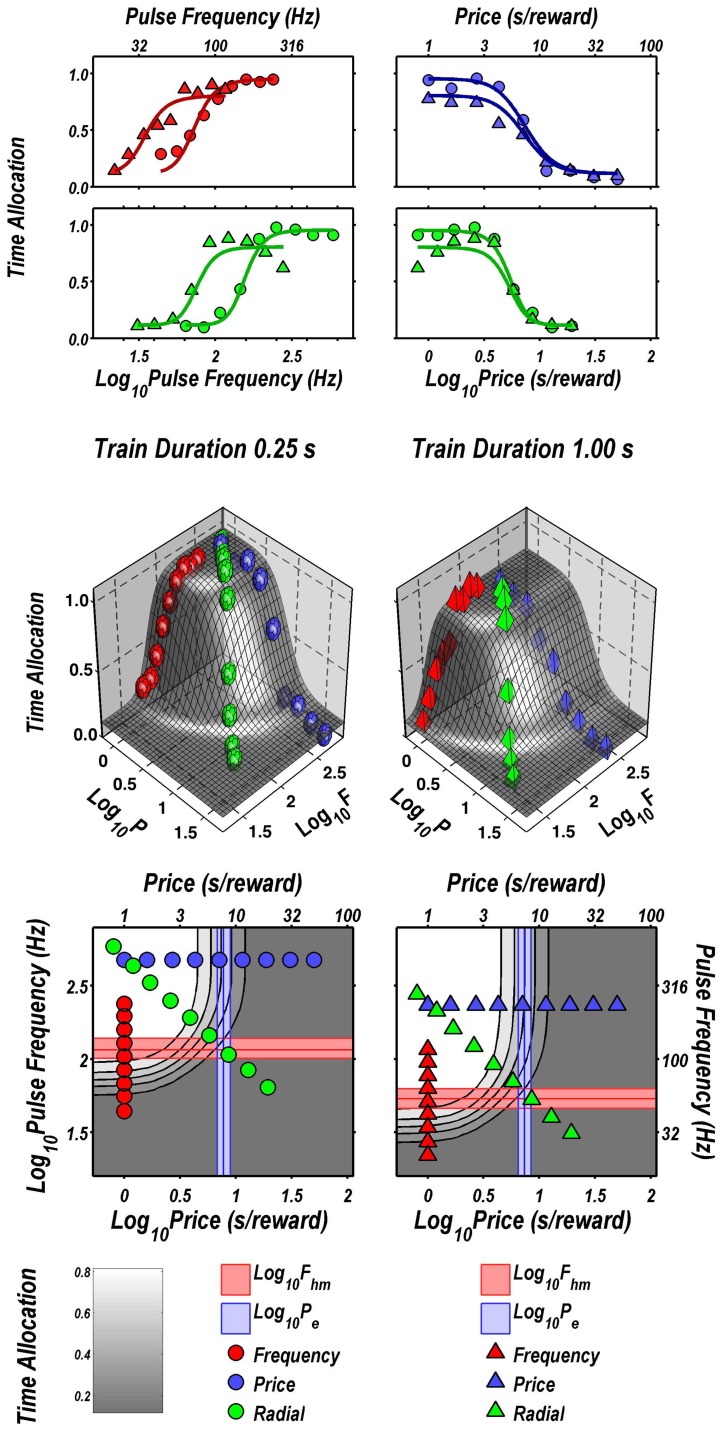
**Sample two- and three-dimensional representations of the data from Rat C26**. The top four panels show the set of “pseudo-sweeps” (see section Behavioral Testing) comprising the reward mountain. **Upper left**: time allocated to reward pursuit as a function of pulse frequency (red circles: 0.25 s duration; red triangles: 1.0 s duration) with price held constant. Shortening the train duration shifts the curve toward higher pulse frequencies. **Upper right**: time allocated to reward pursuit as a function of price with pulse frequency held constant at a high value. This is one of the three cases in which changes in train duration did not alter the position of the mountain along the price axis (blue circles: 0.25 s duration; blue triangles: 1.0 s duration). **Second row:** time allocated to reward pursuit as a function of conjoint variation in both pulse frequency and price (green circles: 0.25 s duration; green triangles: 1.0 s duration). Time allocation along the radial pseudo-sweep is plotted against pulse frequency (left panel) and price (right panel). **Third row**: 3D surfaces showing how time allocation varies as a function of pulse frequency and price. Individual data means along the pulse-frequency (red), price (blue), and radial (green) sweeps are designated by polyhedrons (0.25 s trains) or pyramids (1.0 s trains). **Bottom row:** contour graphs corresponding to the surfaces in the row above. The vertical blue line represents the estimate of *P*_*e*_, and the surrounding band represents the corresponding 95% confidence interval. The horizontal red line represents the estimate of *F*_*hm*_, and the surrounding band represents the corresponding 95% confidence interval.

Figure 2 illustrates the fit of the surface described by Equation 2 to the data for one subject (Y12). The upper four panels depict two-dimensional (2D) sections through the fitted surface. Time allocation at the 1 s (triangles) and 0.25 s (circles) train durations are shown as a function of pulse frequency (red), price (blue), or both (green) along with projections of the fitted surface. Thus, the red points constitute pulse-frequency pseudo-sweeps, the blue points constitute price pseudo-sweeps, and the green points, shown in two orthogonal views, constitute radial pseudo-sweeps. Below the 2D plots, the data from all three pseudo-sweeps are combined in 3D scatter plots along with wire-mesh depictions of the fitted surfaces. The contour plots at the bottom provide a collapsed topographic view of the surface from above; the curved lines are the perimeters of horizontal sections cut at 10% increments in time allocation. The red, blue, and green symbols depict the trajectories of the pulse-frequency, price, and radial pseudo-sweeps, respectively. Also shown in the bottom panels are the values of the position parameters, *P*_*e*_ and *F*_*hm*_, and their surrounding 95% confidence regions (blue vertical lines surrounded by light-blue shading and red horizontal lines surrounded by light-red shading, respectively). In what follows, we use the contour maps to capture the displacement of the mountain along the orthogonal directions of price (a shift in *P*_*e*_) and pulse frequency (a shift in *F*_*hm*_).

**Figure 3:**
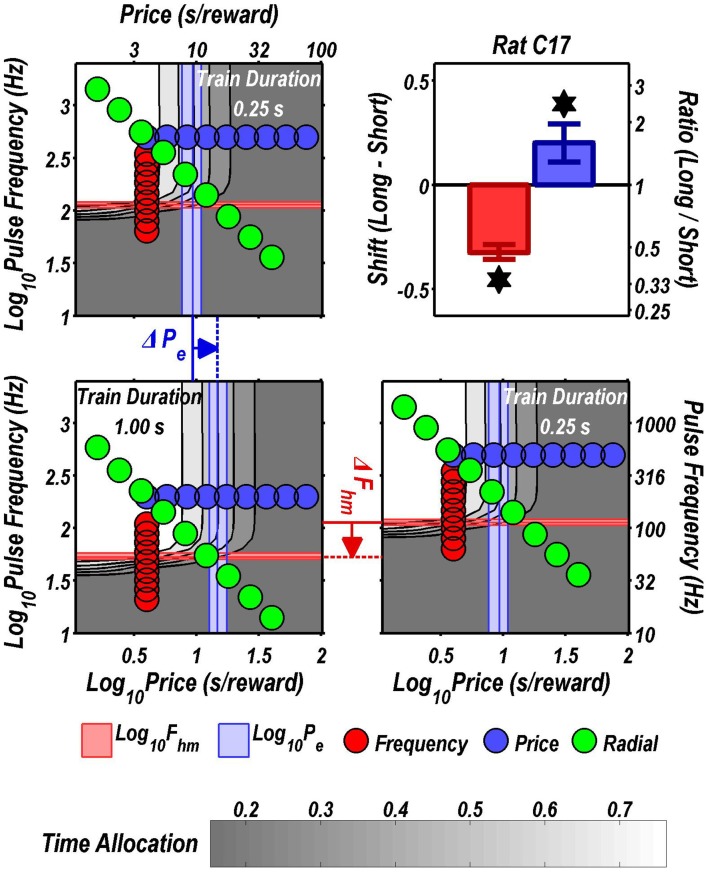
**Shifts of the reward mountain**. The contour graph for the long-duration (1 s) train is shown in the lower left. For comparison the contour graph for the short-duration (0.25 s) train is shown twice, in the upper left and lower right. The *P*_*e*_ parameter determines the location along the price axis, whereas the *F*_*hm*_ parameter determines the location of the mountain along the pulse-frequency axis. The vertical blue line represents the estimate of *P*_*e*_, and the surrounding band represents the corresponding 95% confidence interval. The horizontal red line represents the estimate of *F*_*hm*_, and the surrounding band represents the corresponding 95% confidence interval. The bar graph shows the mean shifts in the location parameters due to the increase in train duration from 0.25 to 1 s along with the associated 95% confidence intervals. ^*^Indicates that the 95% confidence interval does not include zero.

Figures 3 through 8 illustrate the shifts resulting from increasing the train duration. The contour graph for the short train duration is plotted twice, in the top left and bottom right corners, whereas the contour graph for the long train duration is plotted once, in the bottom left hand side. This format makes readily apparent any shifts in *P*_*e*_ (top to bottom comparison) and *F*_*hm*_ (left to right comparison). The magnitudes of the shifts in the *P*_*e*_ and *F*_*hm*_ parameters are represented in the bar graphs in the upper-right panel of each figure. Each bar represents the median difference between the 1000 estimates of a location parameter for the short and long train duration conditions; the 95% bootstrap confidence intervals represent the 2.5 and 97.5 percentiles of these 1000 differences. The difference between the median estimate for the short and long train duration was considered statistically reliable when the associated confidence region did not include 0.

**Figure 4:**
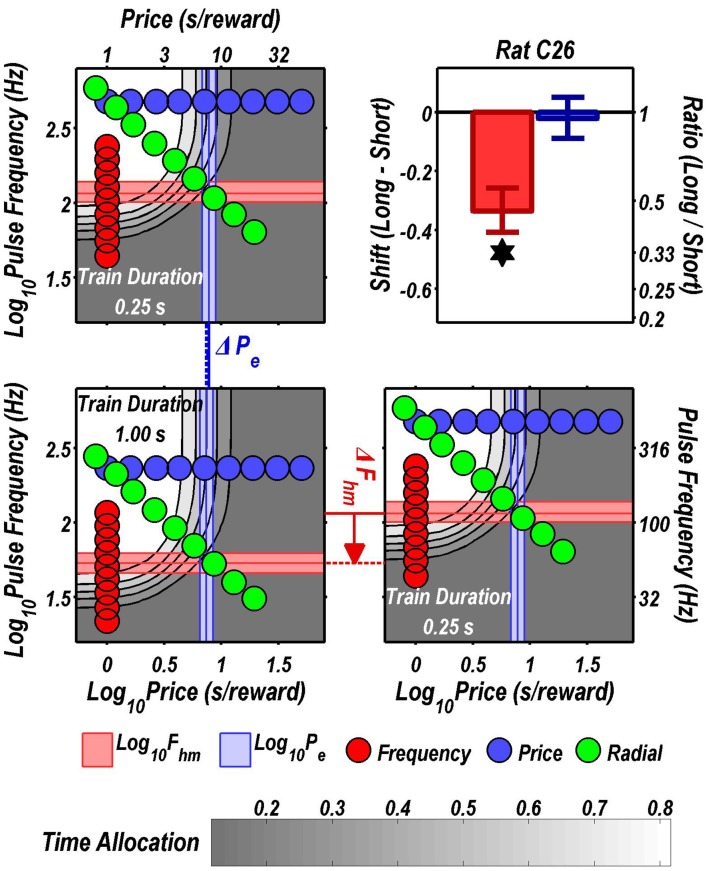
**Shifts of the reward mountain**. The contour graph for the long-duration (1 s) train is shown in the lower left. For comparison the contour graph for the short-duration (0.25 s) train is shown twice, in the upper left and lower right. The *P*_*e*_ parameter determines the location along the price axis, whereas the *F*_*hm*_ parameter determines the location of the mountain along the pulse-frequency axis. The vertical blue line represents the estimate of *P*_*e*_, and the surrounding band represents the corresponding 95% confidence interval. The horizontal red line represents the estimate of *F*_*hm*_, and the surrounding band represents the corresponding 95% confidence interval. The bar graph shows the mean shifts in the location parameters due to the increase in train duration from 0.25 to 1 s along with the associated 95% confidence intervals. ^*^Indicates that the 95% confidence interval does not include zero.

**Figure 5:**
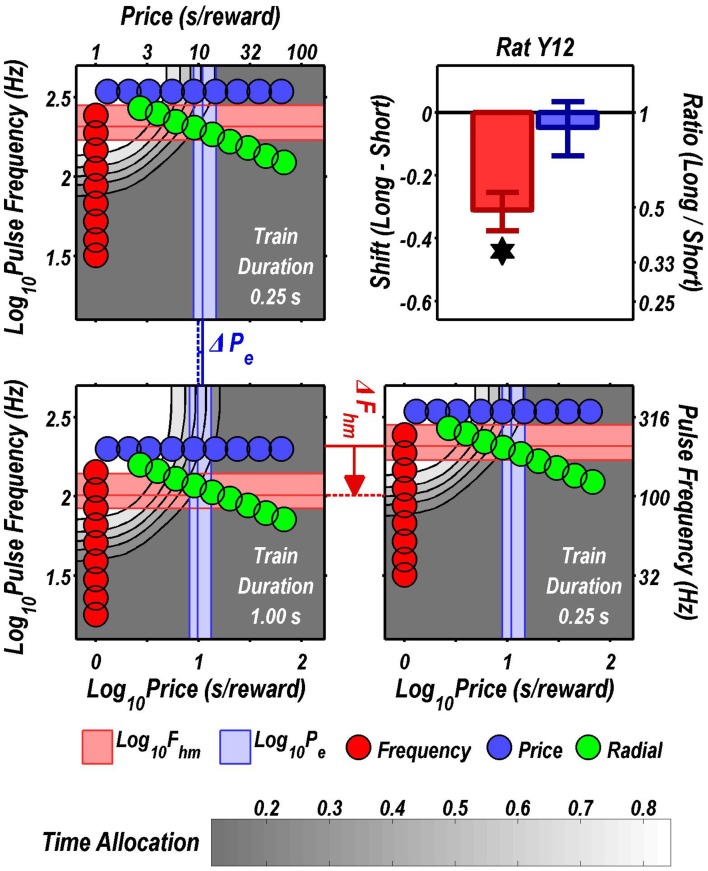
**Shifts of the reward mountain**. The contour graph for the long-duration (1 s) train is shown in the lower left. For comparison the contour graph for the short-duration (0.25 s) train is shown twice, in the upper left and lower right. The *P*_*e*_ parameter determines the location along the price axis, whereas the *F*_*hm*_ parameter determines the location of the mountain along the pulse-frequency axis. The vertical blue line represents the estimate of *P*_*e*_, and the surrounding band represents the corresponding 95% confidence interval. The horizontal red line represents the estimate of *F*_*hm*_, and the surrounding band represents the corresponding 95% confidence interval. The bar graph shows the mean shifts in the location parameters due to the increase in train duration from 0.25 to 1 s along with the associated 95% confidence intervals. ^*^Indicates that the 95% confidence interval does not include zero.

**Figure 6:**
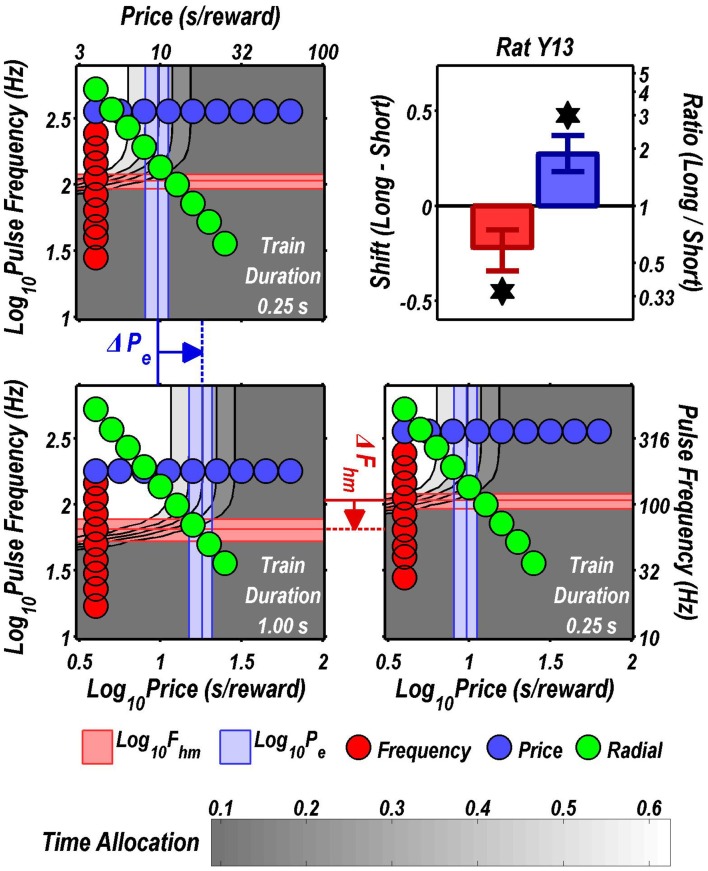
**Shifts of the reward mountain**. The contour graph for the long-duration (1 s) train is shown in the lower left. For comparison the contour graph for the short-duration (0.25 s) train is shown twice, in the upper left and lower right. The *P*_*e*_ parameter determines the location along the price axis, whereas the *F*_*hm*_ parameter determines the location of the mountain along the pulse-frequency axis. The vertical blue line represents the estimate of *P*_*e*_, and the surrounding band represents the corresponding 95% confidence interval. The horizontal red line represents the estimate of *F*_*hm*_, and the surrounding band represents the corresponding 95% confidence interval. The bar graph shows the mean shifts in the location parameters due to the increase in train duration from 0.25 to 1 s along with the associated 95% confidence intervals. ^*^Indicates that the 95% confidence interval does not include zero.

**Figure 7:**
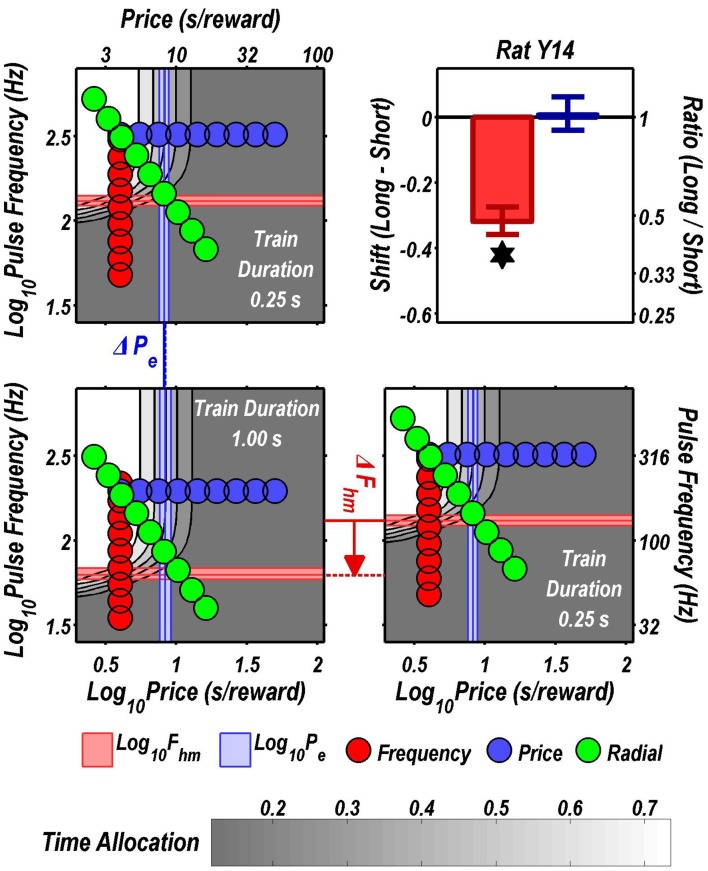
**Shifts of the reward mountain**. The contour graph for the long-duration (1 s) train is shown in the lower left. For comparison the contour graph for the short-duration (0.25 s) train is shown twice, in the upper left and lower right. The *P*_*e*_ parameter determines the location along the price axis, whereas the *F*_*hm*_ parameter determines the location of the mountain along the pulse-frequency axis. The vertical blue line represents the estimate of *P*_*e*_, and the surrounding band represents the corresponding 95% confidence interval. The horizontal red line represents the estimate of *F*_*hm*_, and the surrounding band represents the corresponding 95% confidence interval. The bar graph shows the mean shifts in the location parameters due to the increase in train duration from 0.25 to 1 s along with the associated 95% confidence intervals. ^*^Indicates that the 95% confidence interval does not include zero.

**Figure 8:**
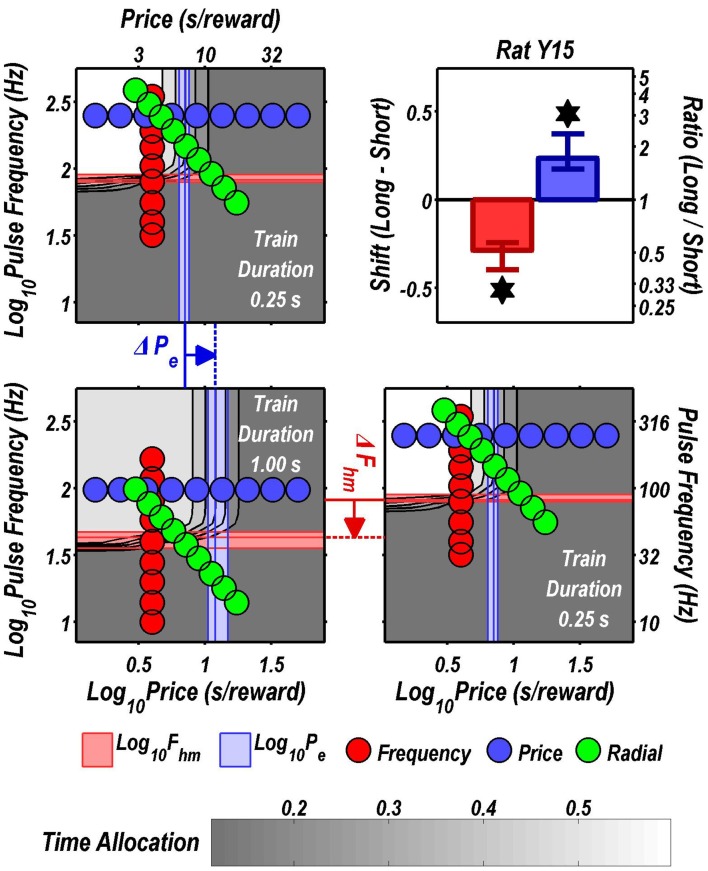
**Shifts of the reward mountain**. The contour graph for the long-duration (1 s) train is shown in the lower left. For comparison the contour graph for the short-duration (0.25 s) train is shown twice, in the upper left and lower right. The *P*_*e*_ parameter determines the location along the price axis, whereas the *F*_*hm*_ parameter determines the location of the mountain along the pulse-frequency axis. The vertical blue line represents the estimate of *P*_*e*_, and the surrounding band represents the corresponding 95% confidence interval. The horizontal red line represents the estimate of *F*_*hm*_, and the surrounding band represents the corresponding 95% confidence interval. The bar graph shows the mean shifts in the location parameters due to the increase in train duration from 0.25 to 1 s along with the associated 95% confidence intervals. ^*^Indicates that the 95% confidence interval does not include zero.

**Figure 9:**
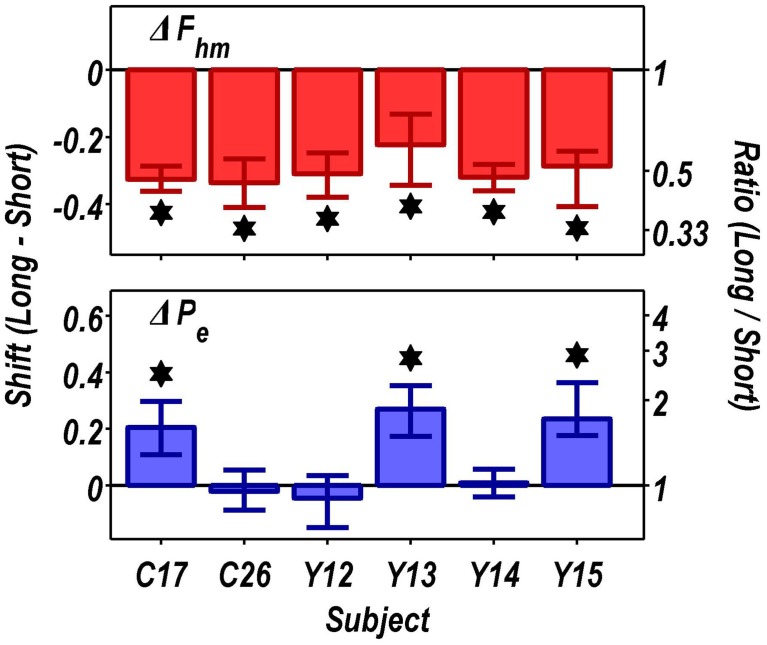
**Summary of the shifts in the position of the reward-mountain produced by increasing the train duration from 0.25 to 1 s**. Increasing the train duration reduced the pulse frequency required to produce a half-maximal reward in all rats (upper panel). In contrast, there was no change, in three cases, in the price at which time allocation for a maximal reward fell halfway between *TA*_max_ and *TA*_min_, whereas lengthening the train duration reliably increased the value of this position parameter in the remaining three rats (lower panel). ^*^Indicates that the 95% confidence interval does not include zero.

Figure 9 summarizes the shifts in the location parameters. Shifts in *F*_*hm*_ (upper panel, red bars) were observed in all six rats tested. These shifts were all in the predicted direction: increasing the train duration decreased the pulse frequencies required to drive subjective reward intensity to half its maximal value. In three cases (rats C26, Y12, Y14), changing the train duration shifted the mountain along the pulse-frequency axis without producing any reliable shift along the price axis (lower panel, blue bars). However, in the remaining three cases (rats C17, Y13, Y15), increasing the duration of the train increased the value of *P*_*e*_.

In all cases, the maximal time allocation attained at the long train duration was reliably shorter than at the short train duration. Typically, this reduction was modest (less than 10%), but in the case of rat Y14, the decrease (27%) was more pronounced.

**Figure 10:**
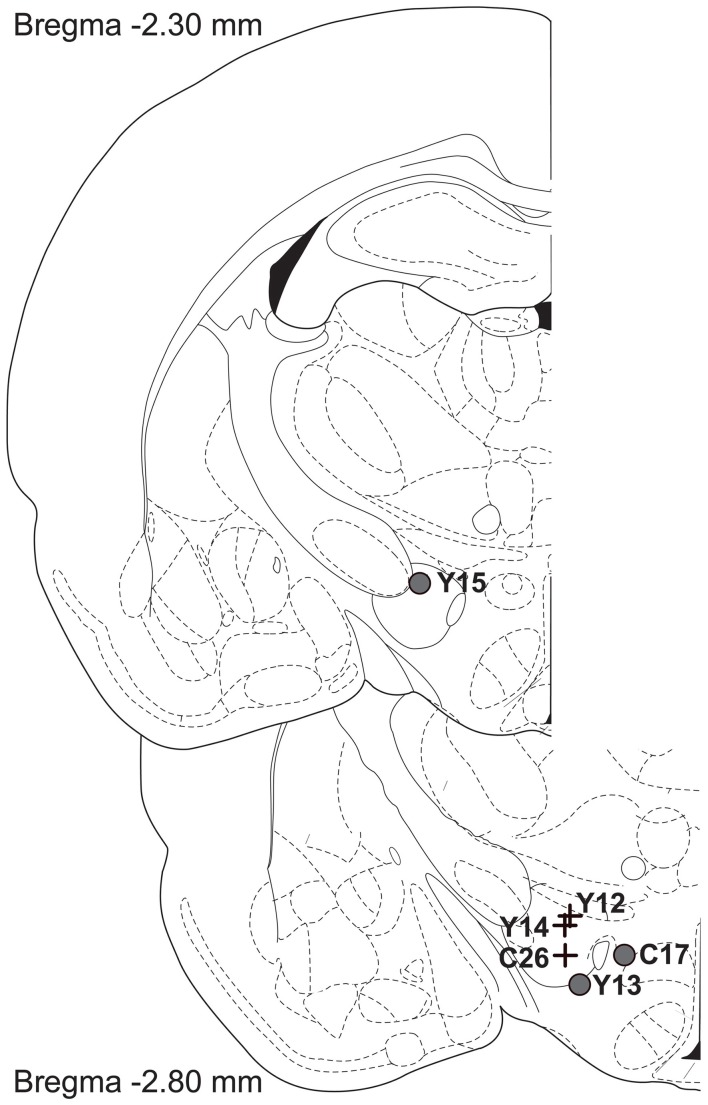
**Location of the electrode tips**. Plus signs designate placements for which the reward mountain did not shift along the price axis, whereas filled circles designate placements for which rightward shifts were observed when the train duration was increased.

Figure 10 shows histological reconstruction of the stimulation sites. The electrode tips for the three cases in which the mountain shifted exclusively along the pulse-frequency axis (C26, Y12, Y14; plus signs) are tightly clustered, whereas the tips for the three cases in which the mountain shifted as well along the price axis (C17, Y13, Y15; filled circles) lie outside the cluster.

## Discussion

Traditional analyses of the effects produced by manipulating brain reward circuitry are two-dimensional: reward-seeking performance is evaluated as a function of a single independent variable, typically the stimulation strength or response cost. The three-dimensional reward-mountain model (Arvanitogiannis and Shizgal, 2008; Hernandez et al., 2010) depicts this long-standing practice from a novel, critical perspective and provides a more informative alternative.

**Figure 11:**
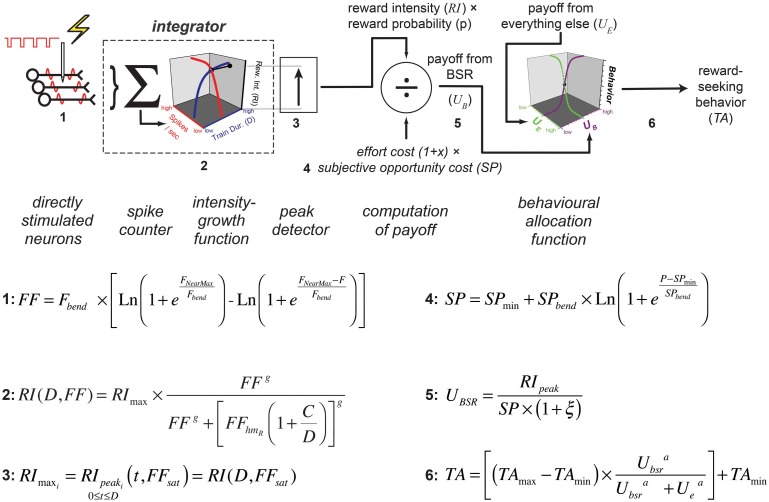
**The single-integrator model**. The version presented here is based on an earlier depiction (Hernandez et al., 2010) but incorporates expressions for the frequency response of the directly stimulated neurons (Equation 4) and for the subjective-price function (Equation 3). The numbering of the graphical elements in the diagram indexes the equations. The following table relates the index numbers in Figure 11 to the equation numbers in the main text and appendix.

According to the reward-mountain model and its antecedents (Gallistel, 1978; Gallistel et al., 1981; Gallistel and Leon, 1991; Simmons and Gallistel, 1994), neural activity induced in the directly-stimulated neurons is translated non-linearly into a stored record of reward intensity. As shown schematically in Figure 11, the payoff from the reward is computed by scalar combination of this information with stored records of the subjective opportunity and effort costs entailed in earning a reward (Solomon et al., 2007; Breton et al., 2009a; Hernandez et al., 2010). The allocation of time to reward pursuit reflects a further non-linear transformation, which combines the subjective estimate of the payoff from the experimenter-controlled reward with an estimate of the payoff from alternate activities, such as grooming, exploring, and resting (Hernandez et al., 2010). This final transformation is based on McDowell's (McDowell, 2005) modification of Herrnstein's Matching Law (Herrnstein, 1970, 1974).

**Table:** 

**Equation number in Figure 11**	**Corresponding manuscript section**	**Manuscript equation number**
1	The Extended Reward Mountain Model	4
2	The dual integrator model, Appendix	5, A.1, A.9
3	Appendix	A.10
4	The Extended Reward Mountain Model	3
5	Appendix	A.4
6	Appendix	A.5

The reward-mountain model highlights how challenging it is to infer, from behavioral data, the stage of processing at which a given experimental manipulation alters performance for a reward. Although the model is a minimal one in the sense that it is hard to see how a simpler formulation could account for the data, it nonetheless entails multiple stages of processing, several of which are non-linear; each stage integrates effects of multiple experimental variables. A given change in the output of the model can thus arise in multiple ways, not all of which may be intuitive.

It follows from the reward-mountain model that the two-dimensional depictions employed in the curve-shift and progressive ratio paradigms are ambiguous and that the conventional interpretations of these depictions do not take sufficient account of the multiple influences on reward-seeking behavior (Arvanitogiannis and Shizgal, 2008; Hernandez et al., 2010; Shizgal and Hernandez, 2010; Shizgal et al., 2012). By measuring and depicting performance as a function of both reward strength and reward cost, the three-dimensional approach derived from the reward-mountain model reduces the ambiguity inherent in the long-used two-dimensional approaches. Of crucial relevance to the present experiment is the ability of the three-dimensional approach, but not the prior two-dimensional methods, to distinguish changes occurring prior to or beyond the output of the “integrator” (Gallistel et al., 1981): the neural circuit that performs temporal and spatial summation of the input from the directly stimulated neurons subserving the rewarding effect.

The reward-mountain model (Hernandez et al., 2010), as well as earlier portrayals of the neural circuitry underlying intracranial self-stimulation (Gallistel, 1978; Gallistel et al., 1981), predict that changes in train duration will alter the stimulation strength required to drive integrator output to a particular level. If more time is available for integration (i.e., if the train duration is longer), and the output of the integrator has not yet reached its asymptote, then a weaker input will suffice to achieve a given level of summation by the end of the train. Therefore, increasing the train duration is predicted to displace the reward mountain toward lower values along the pulse-frequency axis.

### Shifts along the pulse-frequency axis

As shown in Figure 9, increasing the train duration indeed displaced the mountain toward lower values along the pulse-frequency axis in all six rats (red bars), as predicted. This was also the case in the four rats tested previously by Arvanitogiannis and Shizgal (Arvanitogiannis and Shizgal, 2008). Thus, this finding was not altered by the methodological improvements incorporated in the present study and in the pharmacological applications of the three-dimensional measurement method (Hernandez et al., 2010, 2012; Trujillo-Pisanty et al., 2011, 2012).

One of the methodological improvements concerns the schedule of reinforcement. In the current study, a “cumulative handling-time” schedule (Breton et al., 2009b) was used. This schedule forces the subject to choose between work (holding down the lever) or leisure (grooming, resting, exploring, etc.); time spent in leisure activities reduces the number of experimenter-controlled rewards that the subject can earn. In contrast, the variable-interval schedule employed in the Arvanitogiannis and Shizgal (2008) study provides weaker experimental control over reward cost. The subject may succeed in harvesting most of the rewards on offer even it presses the lever only intermittently. In between these work bouts, the subject can engage in leisure activities without much consequent loss of income.

A second methodological improvement is the use of random rather than sequential sampling of the independent variables. We have shown previously (Breton et al., 2009b) that when reward cost remains constant during repeated “sweeps” of the pulse frequency, performance becomes insensitive to the cost variable; sensitivity is restored when both variables are sampled randomly, as was done here.

The third methodological improvement is the application of resampling methods (Efron and Tibshirani, 1994) in fitting the mountain model and estimating its parameters. The observed verification of the prediction that the mountain will be displaced along the pulse-frequency axis constitutes a partial validation of the improved methods.

### Shifts along the price axis

The reward-mountain model makes a negative prediction as well as a positive one concerning the effect of varying the train duration. The positive prediction, displacement toward lower values along the pulse-frequency axis as the train duration is increased, was borne out by the results from all six rats. In contrast, the negative prediction was borne out by the results from only three subjects.

The negative prediction of the model is that the mountain will not move along the price axis when train duration is changed. This prediction arises from the ability of changes in stimulation strength to compensate for changes in temporal integration. In the model depicted in Figure 11, the train duration does not change the maximum reward intensity attainable; instead it alters the stimulation strength required to drive reward intensity to a given level. Once the stimulation strength has been suitably adjusted for a decrease in train duration, performance will be restored to the level observed at the longer duration, and no alteration in price will be required. This prediction was borne out in the results from rats C26, Y12, and Y14. However, contrary to the prediction, displacements along the price axis were observed in the results from rats C17, Y13, and Y15 (Figure 9). The detection of such shifts in the present study, but not in the prior work by Arvanitogiannis and Shizgal (Arvanitogiannis and Shizgal, 2008), may be a consequence of across-study differences in electrode placement or of the methodological improvements embodied in the current design, which shrank the confidence intervals around the estimates of the parameter controlling the location of the mountain along the x-axis.

### The dual-integrator model

The displacements along the price axis can be accounted for by relaxing the assumption of a homogeneous reward substrate. What if the rewarding effect of stimulating certain brain sites arises from the direct activation of a heterogeneous neural population that provides input to multiple integrators? This idea of a heterogeneous substrate has figured prominently in attempts to explain otherwise perplexing data from experiments on the effects of energy-balance manipulations (Blundell and Herberg, 1968; Carr and Wolinsky, 1993; Fulton et al., 2000, 2006; Shizgal et al., 2001) and lesions (Arvanitogiannis et al., 1996; Waraczynski, 2006) on performance for brain stimulation reward. In this regard, it is interesting to note, in Figure 10, the separation between the electrode tips in the subjects demonstrating shifts along the price axis (plus signs) and the subjects showing shifts uniquely along the pulse-frequency axis (circles). We have explored, by means of simulations and additional surface fits, how the multiple-integrator idea might account for the shifts along the price axis, and we propose a way to test this hypothesis.

In the reward-mountain model, *F*_*hm*_, the frequency required to drive reward intensity to half of its maximal value, varies as a declining, rectangular, hyperbolic function of the train duration (Gallistel, 1978; Sonnenschein et al., 2003; Hernandez et al., 2010):

(5) $$FF_{hm}(D)=FF_{hm_{R}}\times \left(1+\frac{C}{D}\right)$$

where

$$\begin{array}{l} FF_{hm}\left(D\right)=\text{the firing frequency required to produce a reward of half-maximal intensity at train duration D}\\ \;FF_{hm_{R}}=\text{the rheobase: the firing frequency required to produce a reward of half-maximal intensity at an infinitely long train duration}\\ \;C=\text{the chronaxie: the train duration at which }FF_{hm}\;\left(D\right)\text{ is twice }FF_{hm_{R}} \end{array}$$

**Figure 12:**
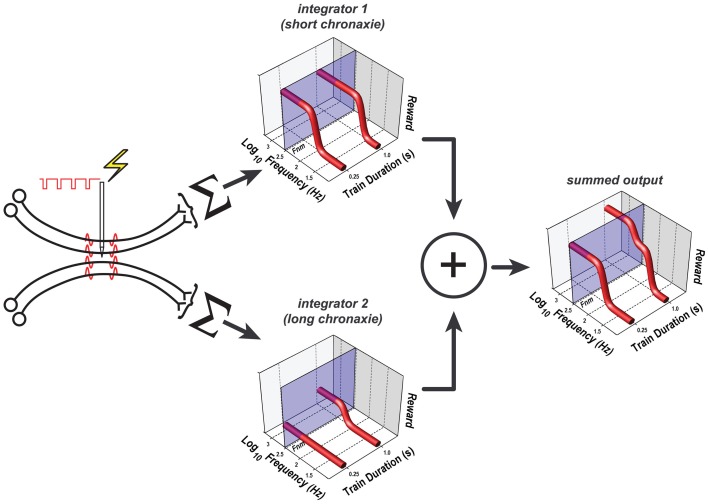
**The dual-integrator model**. Only the components that differ from those in Figure 11 are shown; the remaining components are common to the single- and dual-integrator models. The rewarding effect arises from the direct activation of two subpopulations of neurons, each of which projects to a different spatio-temporal integrator; the weighted outputs of these two integrators are pooled. One integrator has a shorter chronaxie than the other, which renders it less sensitive to the reduction of the train duration from 1 to 0.25 s. Note that the pulse-frequency axis is inverted: the frequency decreases from left to right. The translucent blue planes are positioned at *F*_Near Max_ (*F*_*nm*_), the pulse frequency beyond which reward intensity approaches asymptote. At both the short and long train durations, the growth of reward intensity at the output of integrator 1 (upper 3D graph) is largely complete at pulse frequencies lower than *F*_Near Max_. Although reward growth at the output of integrator 2 is also largely complete at pulse frequencies lower than *F*_Near Max_ when the train duration is 1 s (lower 3D graph), reward growth has not yet begun at this pulse frequency when the train duration is 0.25 s. Thus, reward fails to grow as a function of pulse frequency when the train duration is short, and the summed output of the two integrators (right-hand 3D graph) is lower at the short train duration than at the long. The parameters used to generate the reward-growth functions in Figure 12 are from the fit of the dual-integrator model to the data from rat C17 (Figures 13, 14).

This function, which has provided a very good fit to data from prior studies (Gallistel, 1978; Sonnenschein et al., 2003), has two parameters. The horizontal asymptote of this function is called the rheobase, whereas the curvature of the function is determined by the chronaxie, the train duration at which *FF*_*hm*_ equals twice the rheobase. Suppose that the electrode in animals C17, Y13, and Y15 evoked a volley of action potentials in two subsets of primary reward neurons, each projecting to a separate integration network with a different chronaxie, rheobase, and maximum reward intensity. The outputs of the two integrators are summed, and the result is then combined with the values of the remaining variables determining reward pursuit, as stipulated by the single-integrator version of the reward-mountain model (e.g., Figure 11). This dual-integrator version of the model and the reward-growth functions it generates are shown in Figure 12; the model is derived in the appendix. This expanded version of the mountain model incorporates empirically supported expressions for the subjective assessment of opportunity cost (Solomon et al., 2007) and for the frequency response of the directly stimulated neurons subserving the rewarding effect (Solomon et al., 2010). The appendix includes a detailed discussion of why even the expanded version of the single-integrator model cannot account for the observed shifts of the mountain along the price axis.

Figure 12 shows that the curve describing the growth of integrator output as a function of pulse frequency at the 1 s train duration is pushed toward higher frequencies when the train duration is reduced to 0.25 s. In contrast, little or no growth in output is observed at the 0.25 s train duration in the case of the integrator with the longer chronaxie (integrator 2). The reason for the absence of reward growth at the 0.25 s train duration is that the pulse frequencies required to produce substantial output from this longer-chronaxie integrator when the train duration is this short exceed the frequency-following capability of the directly stimulated neurons. Thus, this integrator can no longer contribute significantly to the summed output, and the maximum summed output achievable declines. The price of the reward must be decreased in compensation, reducing the value of the *P*_*e*_ parameter.

**Figure 13:**
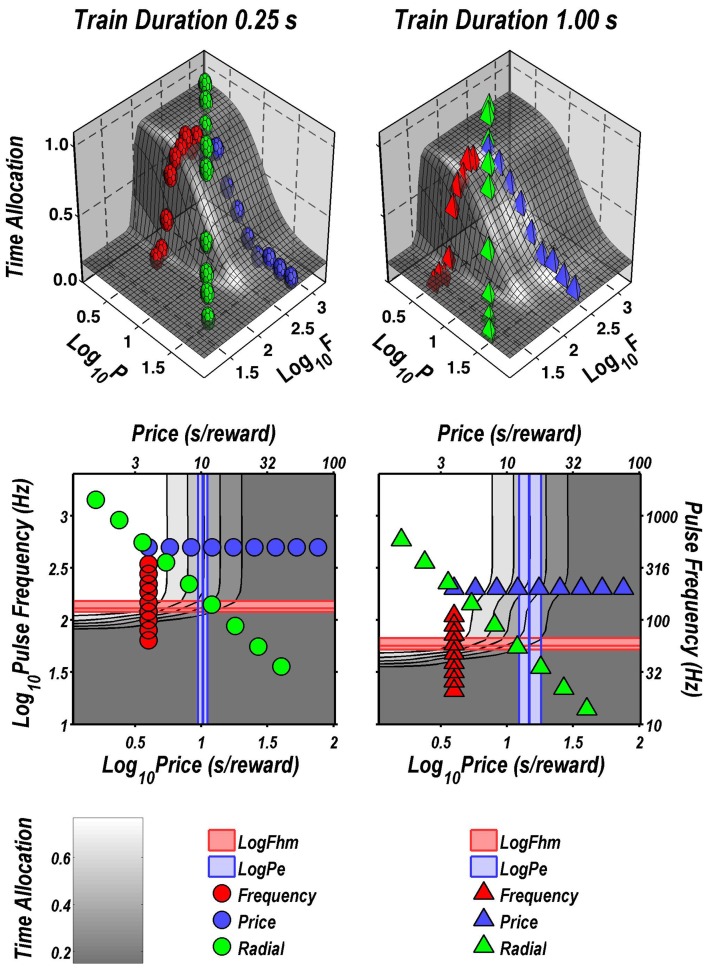
**Fit of the dual-integrator model to the data from rat C17. Top row**: 3D surfaces showing how time allocation varies as a function of pulse frequency and price. Individual data means along the pulse-frequency (red), price (blue), and radial (green) sweeps are designated by polyhedrons (0.25 s trains) or pyramids (1 s trains). Note the indentation in the surface fit to the data obtained at the longer train duration (upper right). This indentation is due to the offset between the pulse frequencies over which reward intensity grows in the two integrators (upper and lower 3D graphs in Figure 12). **Bottom row:** contour graphs corresponding to the surfaces in the row above. The vertical blue line represents the estimate of *P*_*e*_, and the surrounding band represents the corresponding 95% confidence interval. The horizontal red line represents the estimate of *F*_*hm*_, and the surrounding band represents the corresponding 95% confidence interval. The wiggles in the contour lines in the graph for the longer train duration (lower right) correspond to the indentation in the surface shown above.

**Figure 14:**
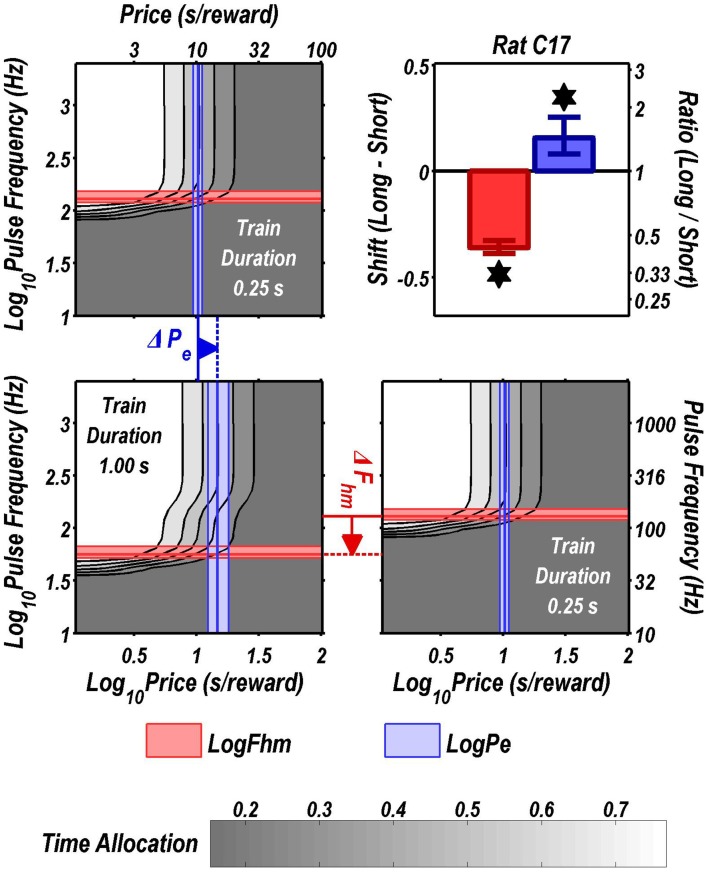
**Shifts of the reward mountain in the fit of the dual-integrator model to the data from rat C17**. The contour graph for the long-duration (1 s) train is shown in the lower left. For comparison the contour graph for the short-duration (0.25 s) train is shown twice, in the upper left and lower right. For clarity, the sampling vectors (the series of points designating the pulse frequencies and prices tested) have been omitted. These can be seen in Figures 3, 13. For comparison with the fit of the single-integrator model to this same dataset, see Figure 3. Note the wiggles in the contour lines for the longer train duration (lower left), which are due to the offset between the pulse frequencies over which reward intensity grows in the two integrators (upper and lower 3D graphs in Figure 12). The *P*_*e*_ parameter determines the location along the price axis, whereas the *F*_*hm*_ parameter determines the location of the mountain along the pulse-frequency axis. The vertical blue line represents the estimate of *P*_*e*_, and the surrounding band represents the corresponding 95% confidence interval. The horizontal red line represents the estimate of *F*_*hm*_, and the surrounding band represents the corresponding 95% confidence interval. The bar graph shows the mean shifts in the location parameters due to the increase in train duration from 0.25 to 1 s. As this figure shows, the dual-integrator model can account for the observed shift of the mountain rightward along the price axis when the train duration is increased from 0.25 to 1 s.

Figures 13, 14 show the results of a constrained fit of the dual-integrator model to the results from rat C17. The constraints were required for two reasons. First, as explained in the appendix, the dual-integrator model adds three parameters to the mountain model: the original *F*_*hm*_ and *P*_*e*_ parameters are replaced by two chronaxie parameters, two rheobase parameters, and a weighting parameter specifying the maximum reward intensity achievable by integrator 1 as a proportion of the summed maxima for the two integrators. One minus this latter value gives the weight for integrator 2. A denser mass of data would be required to estimate the values of this larger number of parameters with the same precision achieved in the fit of the single-integrator model. Second, good estimates of the chronaxie and rheobase parameters require that one or more additional train durations be tested and that these include a value at which the function has approached rheobase. Neither of these requirements are met by the existing dataset and thus additional constraints were required. The values of the *a*, *g*, *TA*_max_ and *TA*_min_ parameters were fixed at the estimates obtained in the initial fits (Figures 3–8), reducing the number of free parameters to be estimated. In addition, an upper limit of 6 s was imposed on the chronaxie parameters. The dual-integrator model derived in the appendix was then fit with these constraints in force, using the resampling approach described above.

Figure 13 shows the 3D surfaces and contour maps generated by the constrained fit of the dual-integrator model to the results from rat C17. The value of the subjective-price and frequency-following parameters were the same as those used in the initial fits, as specified in section The Extended Reward Mountain Model. Given these values, both integrators contribute to the rewarding effect at the longer (1 s) train duration, but the contribution of the integrator with the higher rheobase and the longer chronaxie drops out at the shorter (0.25 s) train duration due to the inability of the directly stimulated neurons to fire at the required pulse frequencies. Due to the composite nature of the reward-growth function at the 1 s train duration, an indentation appears in the surface. The reason for this indentation and the corresponding wiggle in the contour graph is that as the pulse frequency is increased, reward growth in integrator 1 approaches asymptote before there is significant reward growth at the output of integrator 2 (see the 3D graph on the right of Figure 12).

Figure 14 shows the contour graphs obtained from the constrained fit of the dual-integrator model, in the same format as the depictions of the fits of the single-integrator model in Figures 3–8. Note that the mountain for the long train duration is shifted rightward with respect to the mountain for the short train duration. This indicates that the rat was willing to pay higher prices for rewards of a given intensity. According to the dual-integrator model, the increase in train duration shifts the reward-growth function for the long-chronaxie integrator (lower 3D graph in Figure 12) sufficiently so as to allow the output of this integrator to approach its maximum before the frequency-following limit is reached. As a result, the summed reward intensity at the 1 s train duration (right-hand 3D graph in Figure 12) rises higher than at the 0.25 s duration, and the rat is willing to pay more for these high-valued “goods.”

The results of the constrained fit argue for the plausibility of the dual-integrator model. How can this hypothesis be tested empirically? One approach would be to obtain a richer dataset that included a train duration sufficiently long to support accurate chronaxie and rheobase estimates. The dual-integrator model could then be pitted against the simpler version of the mountain model and the results adjudicated by means of the Akaike or Bayes information criterion. A more direct test could be performed in a dual-operant paradigm. According to the dual-integrator model, the maximum reward value achieved in cases such as those shown in Figures 12–14 is lower at the short train duration than at the long duration. If the pulse frequency were set to a value sufficiently high to achieve asymptotic reward growth (i.e., at a value at which the contour lines in Figures 13, 14 run vertically), the rat should prefer the long-duration train over the short-duration train.

With only six observations in hand, one cannot draw firm conclusions from the locations of the electrode tips (Figure 10). That said, it is of interest that the three stimulation sites at which shifts along the price axis were noted are separated from the three tightly clustered sites at which no such shifts were seen. If such a pattern were also seen in future tests, this would support the notion of a heterogeneous reward substrate that is sampled differentially as a function of the location of the electrode tip (Fulton et al., 2006).

## Conclusions

The reward-mountain model and the associated measurement method have advanced efforts to pin down the stage of processing at which psychomotor stimulants (Hernandez et al., 2010, 2012), neuroleptics (Trujillo-Pisanty et al., 2012), and cannabinoids (Trujillo-Pisanty et al., 2011) act to alter performance for BSR. These tools promise to produce similar benefits in the analysis of effects produced by other drugs as well as by manipulations such as lesions, alterations in energy balance, and optogenetic activation or silencing. That said, the results of the current study both sound a note of caution and offer a remedy concerning the interpretation of results obtained by means of the 3D method. The dual-integrator model developed here shows that a manipulation that acts prior to the output of the integrator, such as changing the train duration, can nonetheless shift the mountain along the price axis under a special condition: when the increase in pulse frequency required to offset the effect of the manipulation outstrips the ability of the directly stimulated neurons to fire in response to each and every stimulation pulse. The use of moderate to high currents in future studies would reduce this risk by moving the value of the *F*_*hm*_ parameter away from *F*_Near Max_, the value that marks the breakdown of perfect frequency following (Solomon et al., 2010).

New optogenetic methods (Yizhar et al., 2011) promise to advance research on brain reward circuitry in many ways, several of which are directly germane to the issues raised here. These methods allow much more specific targeting of neural activation, which should help identify the components of the BSR substrate. Once this has been done, models such as the one proposed here could be put to exacting tests by optogenetic activation or silencing of specific circuit elements. Such methods have already identified multiple promising candidates for the directly stimulated stage of the circuitry subserving medial forebrain bundle self-stimulation (Jennings et al., 2013; Kempadoo et al., 2013), which lends plausibility to the notion of multiple integrators.

Gallistel has long argued that the output of the integrator is recorded in an enduring memory of reward intensity (Gallistel et al., 1974, 1981). Such memories are believed to play crucial roles in reward-seeking behaviors, both salubrious and pathological. Working out the structure of the circuitry subserving spatio-temporal integration of reward signals should shed light on how such memories are formed. Along with stored information about the costs, risks, and kinds of rewards available (Shizgal, 1997), a record of reward intensity provides essential data for the processes that select goals and allocate behavior to their pursuit.

### Conflict of interest statement

The authors declare that the research was conducted in the absence of any commercial or financial relationships that could be construed as a potential conflict of interest.

## Appendix

### The original single-integrator version of the reward-mountain model

A paper by Hernandez et al. (2010) derives the version of the reward-mountain model that is extended here. The dependent behavioral variable, time allocation (*TA*), is expressed as a function of two independent variables, pulse frequency (*F*) and price (*P*). (Recall that price is the cumulative time that the rat must depress the lever in order to trigger a reward.) The extensions proposed here incorporate two transformations that allow the independent variables to be treated in a more realistic fashion than in the earlier versions of the model. The stimulated neurons are no longer assumed to be capable of firing in response to every stimulation pulse, regardless of the pulse frequency. When the price is trivially small, a modest proportional change of a given magnitude is no longer assumed to have the same behavioral consequence as the same proportional change at higher prices. (For example, in the version proposed here, increasing the price from 0.01 to 0.02 s has a negligible effect, but increasing it from 10 to 20 s has a large effect. In contrast, the effects of both increases are equivalent in the earlier versions.) The extended mountain model is illustrated in Figure 11.

#### The frequency roll-off, reward-growth, and train strength-duration functions

The extension described by Equation 4 (section The Extended Reward Mountain Model; Figure 1, lower panel) translates the pulse frequency into the stimulation-induced spike rate in the directly stimulated neurons responsible for the rewarding effect (Figure 1, right panel).

$$FF=F_{\text{bend}}\times \left[\text{Ln}\left(1+e^{\frac{F_{\text{NearMax}}}{F_{\text{bend}}}}\right)-\text{Ln}\left(1+e^{\frac{F_{\text{NearMax}}-F}{F_{\text{bend}}}}\right)\right]$$

where

$$\begin{array}{l} \;F_{\text{bend}}=\text{parameter governing the abruptness of the transition between the rising and flat segments of the function}\\ F_{\text{NearMax}}=\text{the midpoint of the transitional region} \end{array}$$

Once the pulse frequency surpasses a critical value, *F*_NearMax_, further increases fail to boost the induced firing rate.

The induced rate of firing is translated into the intensity of the rewarding effect by a logistic “reward-growth” function (Sonnenschein et al., 2003; Arvanitogiannis and Shizgal, 2008; Hernandez et al., 2010) based on scaling work carried out by Gallistel's group (Gallistel and Leon, 1991; Simmons and Gallistel, 1994):

(A.1) $$RI(D,FF)=RI_{\max}\times \frac{FF^{g}}{FF^{g}+{\left[FF_{hm}(D)\right]}^{g}}$$

where

$$\begin{array}{l} \;D=\text{duration of the stimulation train}\\ \;FF=\text{induced firing frequency in the directly stimulated neurons}\\ \;FF_{hm}=\text{induced firing frequency that produces a half-maximal reward intensity}\\ \;g=\text{the intensity-growth exponent}\\ \;RI=\text{reward intensity, and}\\ RI_{\max}=\text{the maximum reward intensity achieveable} \end{array}$$

A rectangular hyperbolic function (Gallistel, 1978; Sonnenschein et al., 2003; Hernandez et al., 2010) translates the train duration (*D*) into the induced firing frequency (*FF*_*hm*_), as specified in Equation 5 (section The Dual Integrator Model).

$$FF_{hm}(D)=FF_{hm_{R}}\times \left(1+\frac{C}{D}\right)$$

where

$$\begin{array}{l} FF_{hm}\left(D\right)=\text{the firing frequency required to produce a reward of half-maximal intensity at train duration D}\\ \;FF_{hm_{R}}=\text{the rheobase: the firing frequency required to produce a reward of half-maximal intensity at an infinitely long train duration}\\ \;C=\text{the chronaxie: the train duration at which }FF_{hm}\left(D\right)\text{ is twice }FF_{hm_{R}} \end{array}$$

At any given train duration, *D*, we can recover *F*_*hm*_, the parameter that locates the reward mountain along the pulse frequency axis, by back-solving Equation 4 (section The Extended Reward Mountain Model) as follows:

(A.2) $$F_{hm}(D)=F_{\text{Near Max}}-F_{\text{bend}}\times \text{Ln}\left(e^{\frac{F_{\text{NearMax}}-FF_{hm}(D)}{F_{\text{bend}}}}+e^{-\frac{FF_{hm}(D)}{F_{\text{bend}}}}-1\right)$$

#### The subjective-price function

The second extension introduced here maps the objective price (*P*) into its subjective equivalent (*SP*). According to this function (section The Extended Reward Mountain Model, Equation 3; Figure 1, upper panel), the subjective price remains nearly constant at a minimal value when the objective price is very low, but once the objective price exceeds a critical value, the subjective price starts to grow and eventually approaches the objective price.

$$SP=SP_{\min}+SP_{\text{bend}}\times \text{Ln}\left(1+e^{\frac{P-SP_{\min}}{SP_{\text{bend}}}}\right)$$

where

$$\begin{array}{l} \;SP_{\min}=\text{minimum subjective price}\\ SP_{\text{bend}}=\text{parameter determining the abruptness of the transition between }SP_{\min}\text{ and the rising portion of the subjective-price function} \end{array}$$

The surface-fitting procedure returns an estimate of *SP*_*e*_, the subjective price at which time allocation for a maximally intense reward falls halfway between maximal and minimal time allocation when frequency following is perfect. This estimate can be transformed into the parameter that locates the reward mountain along the price axis, *P*_*e*_, by back-solving the subjective-price equation:

(A.3) $$P_{e}=SP_{\min}+SP_{\text{bend}}\times \text{Ln}\left(e^{\frac{SP_{e}-SP_{\min}}{SP_{\text{bend}}}}-1\right)$$

#### The payoff and behavioral-allocation functions

The payoff from BSR is modeled (Hernandez et al., 2010) as a scalar combination of the strength and cost of the reward:

(A.4) $$U_{bsr}=\frac{RI}{SP\times \left(1+\xi \right)}$$

where *U*_*bsr*_ = the payoff from BSR and

1 + ξ = the subjective rate of exertion entailed in holding down the lever.

The behavioral-allocation function in the reward-mountain model (Hernandez et al., 2010) relates time allocated to depressing the lever to the relative payoffs obtained from BSR and from alternate activities such as grooming, exploring and resting:

(A.5) $$TA=\left[\left(TA_{\max}-TA_{\min}\right)\times \frac{U_{bsr}^{\;a}}{U_{bsr}^{\;a}+U_{e}^{\;a}}\right]+TA_{\min}$$

where

$$\begin{array}{l} \;a=\text{the payoff-sensitivity exponent}\\ \;U_{e}=\text{the payoff from}“\text{everything else}”\;(\text{alternate activities})\\ TA_{\max}=\text{maximum time allocation, and}\\ TA_{\min}=\text{minimum time allocation} \end{array}$$

It follows from Equation A.3, that *TA* will fall mid-way between *TA*_max_ and *TA*_min_ (*TA* = *TA*_mid_) when the payoff from BSR equals the payoff from “everything else” (*U*_*bsr*_*TA*_mid___ = *U*_*e*_). Thus, we can define a subjective price, *SP*_*e*_, such that

(A.6) $$U_{e}=U_{bsr_{TA_{\text{mid}}}}=\frac{RI_{\max}}{SP_{e}\times \left(1+\xi \right)}$$

where

$$\begin{array}{l} SP_{e}=\text{the subjective price at which time allocation for a maximal BSR falls halfway between }TA_{\max}\text{ and }TA_{\text{min}}\text{, and}\\ \;1+\xi =\text{the subjective rate of exertion required to hold down the lever} \end{array}$$

Substituting for *U*_*bsr*_ and *U*_*e*_ in Equation A.5 and dividing the payoff terms by $\frac{RI_{\max}}{SP_{e}\times \left(1+\xi \right)}$, we obtain:

(A.7) $$TA=\left[\left(TA_{\max}-TA_{\min}\right)\times \frac{{\left(\frac{RI}{RI_{\max}}\right)}^{a}}{{\left(\frac{RI}{RI_{\max}}\right)}^{a}+{\left(\frac{SP}{SP_{e}}\right)}^{a}}\right]+TA_{\min}\;\;\;\;\;\;\;$$

This is the equation that was fit to the data to produce the graphs in Figures 2–8.

#### Shifts exclusively along the pulse-frequency axis

We are now in a position to see why, in the single-integrator version of the mountain model (Figure 11), changing the train duration moves the mountain along the pulse-frequency axis and not along the price axis, provided that perfect frequency following doesn't break down. Changing the train duration alters the pulse frequency required to drive reward intensity to its maximal value. Once *RI*_max_ is attained, $\frac{RI}{RI_{\max}}$ equals 1, and *TA* will fall halfway between its minimum and maximum values when *SP* = *SP*_*e*_, regardless of the pulse frequency required to reach *RI*_max_.

#### Shifts along both the pulse-frequency and price axes

The extension of the reward-mountain model allows us to predict what will happen when perfect frequency following does break down. In that case, changing the train duration ***can*** shift the mountain along the price axis as well as along the pulse-frequency axis.

To accommodate the limited frequency-following capability of the stimulated neurons, let us redefine *SP*_*e*_ as *SP*_*e*_*PFF*__, where “PFF” stands for “perfect frequency following.” As long as perfect frequency following is maintained, the “middle” contour of the mountain, the one representing the *TA*_*mid*_ “altitude” (half-way between *TA*_min_ and *TA*_max_), will asymptote at *SP*_*e*_*PFF*__. This follows from Equation A.6, which defines *SP*_*e*_ (and by extension, *SP*_*e*_*PFF*__) as the price at which time allocation to pursuit of a half-maximal reward equals *TA*_mid_. However, if *F*_*hm*_ is near or greater than *F*_Near Max_, then reward intensity can no longer reach its maximum value. In order to achieve a time allocation of *TA*_mid_ for this weaker reward, the price must be decreased. As a result, the middle contour line will start to run vertically at an SP value (and corresponding objective price) lower than *SP*_*e*_*PFF*__ that we will call *SP*_*e*_*VMC*__, where “VMC” represents the vertical orientation of the *TA*_mid_ contour at the highest pulse frequencies. Thus, the mountain shifts leftwards along the price axis.

$$\begin{array}{l} \text{Let }F_{HiF}=\text{the highest pulse frequency the rat can tolerate}\\ \;FF_{HiF}=\text{the corresponding spike rate, and}\\ \;RI_{HiF}=\text{the corresponding reward intensity} \end{array}$$

Given these definitions and Equation A.1,

(A.8) $$SP_{e_{VMC}}=SP_{e_{PFF}}\times \frac{RI_{HiF}}{RI_{\max}}$$

When *F*_*hm*_ « *F*_NearMax_, even at the shorter train duration, then $\frac{RI_{HiF}}{RI_{\max}}$ will approach unity, and *SP*_*e*_*VMC*__ ≈ *SP*_*e*_*PFF*__. As a result, decreasing the train duration will not shift the mountain along the price axis. In contrast, if $F_{hm}\bar{\ll }F_{\text{NearMax}}$ at the short train duration, then *SP*_*e*_*VMC*__ < *SP*_*e*_*PFF*__, and the mountain shifts leftwards along the price axis.

This section of the appendix sounds a note of caution concerning the interpretation of shifts in the position of the reward mountain. The extension of the model to accommodate realistic frequency-following capabilities in the directly stimulated substrate shows how leftward shifts along the price axis could result from treatments, such as drug administration, lesions, or optogenetic manipulations that cause large increases in *F*_*hm*_. In such cases, the observed values of *F*_*hm*_ should be evaluated carefully so as to estimate the ability of the directly stimulated neurons to follow the tested pulse frequencies with good fidelity (Simmons and Gallistel, 1994; Solomon et al., 2010).

Could failure of frequency following in a single-integrator model account for the shifts along the price axis observed in the cases of rats C17, Y13, and Y15 when the train duration was reduced from 1 to 0.25 s? Inspection of Figures 3, 6, 8 suggests that this is highly unlikely: the *F*_*hm*_ values are only 113, 107, and 82 pulses per second, well within the range of high-fidelity frequency following (Simmons and Gallistel, 1994; Solomon et al., 2010). Thus, the single-integrator account cannot plausibly explain observed shifts along the price axis, even when the frequency-following limitation is incorporated in the model. In the next section, we develop a dual-integrator version of the reward-mountain model, and we show in section The Dual Integrator Model that this version does provide a plausible, testable account.

### The dual-integrator version of the reward-mountain model

The distinguishing features of the dual-integrator model are illustrated in Figure 12, which replaces the leftmost components of the single-integrator model shown in Figure 11. The electrode samples from two intermingled populations of fibers, and the synaptic drive produced in each population is integrated separately; the outputs of the two integrators are summed. To accommodate these changes and to make the dependence of *FF*_*hm*_ on *D* explicit, Equation A.1 has been modified as follows:

(A.9) $$RI(D,FF)=\sum \left(RI_{max_{i}}\times \frac{FF^{g}}{FF^{g}+{\left[FF_{hm_{R_{i}}}\left(1+\frac{C_{i}}{D}\right)\right]}^{g}}\right)\;$$

where

                   i∈{integrator 1, integrator 2}

Now, each integrator can achieve a different maximum reward intensity (*RI*_max_*i*__), which is achieved at the end of the pulse train:

(A.10) $$\underset{0\leq t\leq D}{RI_{\max_{i}}=RI_{peak_{i}}\left(t,FF_{sat}\right)}=RI(D,FF_{sat})$$

where

$$FF_{sat}=\text{the frequency of firing that saturates (maximizes) the growth of reward intensity}$$

These maximal, peak values scale the growth of reward intensity at the output of each integrator and thereby weight the contribution of each integrator to the summed output:

(A.11) $$w_{i}=\frac{RI_{\max_{i}}}{\sum RI_{\max_{i}}}$$

where *w*_*i*_ = the relative contribution of integrator *i* to the pooled output when frequency following is perfect.

Equation A.11 expresses the weights in terms of the proportion of the maximum summed output each integrator can contribute when perfect frequency following obtains. By definition, the two weights sum to one:

$$\sum w_{i}=1$$

Generalizing from section Shifts Along Both the Pulse-Frequency and Price Axes we define *RI*_*HiF*_*i*__ as the reward intensity achieved by integrator *i* at the highest pulse frequency that can be tested. When frequency following is perfect, *RI*_*HiF*_*i*__ = *RI*_max_*i*__. Substituting for *RI*_max_*i*__ in Equations A.9 and A.11 and rearranging terms, we obtain:

(*A*.12) $$\frac{RI(D,FF_{HiF})}{RI_{\text{max}}}=\sum \text{​}\left(w_{i}\times \frac{FF_{HiF}^{\;g}}{FF_{HiF}^{\;g}+{\left[FF_{hm_{R_{i}}}\left(1+\frac{C_{i}}{D}\right)\right]}^{g}}\right)$$

If frequency following in the input to either integrator is imperfect, then $\frac{RI(D,FF_{HiF})}{RI_{\max}}<1$. Equation A.12 shows that the higher the values of *FF*_*hm*_*R*__ and C, the more likely that *F*_*hm*_*i*__ (*D*) will approach *F*_NearMax_, and the further *RI*(*D*, *FF*_*HiF*_) will fall below *RI*_max_*i*__. The shorter the train duration, the greater the separation between the pulse-frequencies over which the outputs of the two integrators grow.

In the example illustrated in Figure 12, the chronaxie and rheobase for integrator 2 are larger than for integrator 1. This is why reward growth begins in earnest at a lower frequency at the output of integrator 1 and why the output of integrator 2 does not begin to rise until the output of integrator 1 is already approaching asymptote. At the longer train duration *F*_*hm*_*i*__ (*D*) is still sufficiently below *F*_NearMax_ that the output of integrator 2 can eventually approach its maximum. However, decreasing the train duration to the shorter value pushes the reward-growth function for integrator 2 sufficiently far to the right that the failure of frequency following prevents the output of that integrator from making a substantial contribution to the pooled output. Thus, the summed value of *RI*_max_ is lower at the shorter train duration than at the long. Equation A.8 then applies:

$$SP_{e_{VMC}}=SP_{e_{PFF}}\times \frac{RI_{HiF}}{RI_{\max}}$$

As in the case described in section 7.1.4, *SP*_*e*_*VMC*__ is pulled leftward, away from *SP*_*e*_*PFF*__, producing a shift along the price axis such as those seen in the data from rats C17, Y13, and Y15.

The value of the *P*_*e*_ parameter was derived from *SP*_*e*_*PFF*__, as specified by Equation A.3. The value of *F*_*hm*_ was computed by solving Equation A.13  and then backsolving for *F*_*hm*_ using Equation A.2.

## Abbreviations

2D: two-dimensional
3D: three-dimensional
*a*: the payoff-sensitivity exponent (Equation 1)
BSR: brain stimulation reward
*F*: pulse frequency
*F*_bend_: parameter governing the abruptness of the transition between the rising and flat segments of the frequency-response function (Equation 4)
*F*_*hm*_: pulse frequency at which reward intensity is half-maximal (Equation 1)
*F*_NearMax_: the midpoint of the transitional range of the frequency-response function (Equation 4)
*FF*: induced frequency of firing in the directly stimulated neurons (Equations 2, 4)
*FF*_*hm*_: induced frequency of firing that produces a reward of half-maximal intensity (Equation 2)
*g*: reward-growth exponent (Equation 1)
*P*: price of the stimulation train (cumulative time the lever must be depressed in order to trigger delivery of a train)
*P*_*e*_: price at which time allocation for a maximally intense reward falls halfway between *TA*_max_ and *TA*_min_ (Equation 1)
pps: pulse per second
*SP*: subjective price of the stimulation train (Equations 2, 3)
*SP*_bend_: parameter determining the abruptness of the transition between *SP*_min_ and the rising portion of the subjective-price function (Equation 3)
*SP*_*e*_: subjective price at which time allocation for a maximally intense reward falls halfway between *TA*_max_ and *TA*_min_ (Equation 2)
*SP*_min_: minimum subjective price (Equation 3)
*TA*: time allocation
*TA*_max_: maximal time allocation (Equations 1, 2)
*TA*_min_: minimal time allocation (Equations 1, 2)
VI: variable interval.
