# Corrigendum: Validation and extension of the reward-mountain model

**DOI:** 10.3389/fnbeh.2014.00310

**Published:** 2014-09-11

**Authors:** Peter Shizgal

**Affiliations:** Psychology/Arts and Science, Center for Studies in Behavioural Neurobiology, Concordia UniversityMontreal, QC, Canada

**Keywords:** intracranial self-stimulation, brain stimulation reward, temporal integration, medial forebrain bundle, operant conditioning

There is a subscripting error in the appendix to the article by Breton et al. (2013). In the sentence on page 16 between Equations A.6 and A.7 that begins

“Substituting for *U*_*bsr*_ and *U*_*e*_ in Equation A.5,”

the specified divisor should be $\frac{RI_{\max}}{SP\times \left(1+\xi \right)}$ and not $\frac{RI_{\max}}{SP_{e}\times \left(1+\xi \right)}$. In other words, there should be no subscript for *SP* in the divisor.

## Conflict of interest statement

The authors declare that the research was conducted in the absence of any commercial or financial relationships that could be construed as a potential conflict of interest.
